# Bioethicists Should Be Helping Scientists Think About Race

**DOI:** 10.1007/s11673-020-10068-x

**Published:** 2021-01-07

**Authors:** Camisha Russell

**Affiliations:** Department of Philosophy, 1295 University of Oregon, Eugene, OR 97403-1295 USA

**Keywords:** Race, Bioethics, Assisted reproductive technologies

## Abstract

In this essay, I argue that bioethicists have a thus-far unfulfilled role to play in helping life scientists, including medical doctors and researchers, think about race. I begin with descriptions of how life scientists tend to think about race and descriptions of typical approaches to bioethics. I then describe three different approaches to race: biological race, race as social construction, and race as cultural driver of history. Taking into account the historical and contemporary interplay of these three approaches, I suggest an alternative framework for thinking about race focused on how the idea of race functions socially. Finally, using assisted reproductive technologies as an example, I discuss how bioethicists and scientists might work together using this framework to improve not only their own but broader perspectives on race.

In what follows, I argue that bioethicists should be helping scientists think about race. In making this argument, I am asking both many bioethicists and many scientists to change their practices. The scientists I am talking about are not social scientists but natural ones, life scientists in particular, including medical doctors and researchers. First, I will explain how I think life scientists tend to think about race and outline what I consider a typical approach to bioethics. Then I will put forward an alternative framework for thinking about race. Finally, using assisted reproductive technologies as an example, I will discuss how bioethicists and scientists might work together to improve not only their own but broader perspectives on race.

I am not a life scientist, but when I think about work in the life sciences on race, I see it as essentially focused on answering questions about what race is. Questions like: Is race real in the scientific sense? What, if any, biological, physiological, or genetic features demarcate different racial categories? What, if any, is the most scientifically accurate way to divide people into racial categories? Answers to these first questions about the nature of race would then be expected to suggest answers to further questions about scientific practices, for example: How, if ever, should a concept of race be incorporated in human research? Or pharmaceutical research? Or medical practice (Roberts 2012; Reardon 2005)?

Of course, many past life scientists are now understood to have produced false or misleading “racial science,” presumably having failed to recognize the influence of the racist assumptions and norms of their day on their thinking and research. And indeed, a small number of scientists still see racial difference as so self-evident that they continue to put forward scientific theories purporting to verify and explain that difference (Reich 2018). Nevertheless, I take the broad consensus among life scientists to be that contemporary biology and genetics cannot give us scientifically the “races” whose existence has been assumed, constructed, and reified socio-historically. Put another way, the major contribution of the contemporary life sciences to fighting racism has been to insist that race (as we know it) is not real.

What I am calling a typical approach to bioethics is focused on attempting to determine which practices are ethically permissible in biomedicine and biomedical research in terms of individually conceived ethical rights, duties, obligations, or prohibitions. The ethical, when centred on the idea of personal rights and freedom, comes to be concerned only with what an individual (or hospital or corporation or research team) may be *permitted* to do, where the limits of ethical permissibility are conceived of only in terms of specific harm to the personal freedom of other individuals. While bioethicists can easily recognize that racist policies or racial discrimination in medical or scientific research and practice are ethically impermissible due to the harm they cause the individuals targeted by the discrimination, they will have little to say about the idea of race itself (Russell 2016).

To explain how and why I urge life scientists and bioethicists to consider a new, different approach, I will need to say more about how I think we should understand the idea of race. I would expect readers to be broadly familiar with two possible conceptions of what race is. First is the view of the aforementioned “racial science,” which takes the racial categories that have been “perceived” and elaborated historically (and the differences associated with those categories) to have their basis in the natural world and, therefore, to be “discoverable” or verifiable by science. This view is widely discredited.

The second is the view of many contemporary social scientists, other academics, and laypeople—that race is socially constructed. To be socially constructed is not necessarily not to be real. Though not a scientifically verifiable product of the natural world, race can be considered real in the same way that money is. With money, societies take items found in nature (precious metals or paper) and assign them an exchange value that, though initially arbitrary, cannot be subsequently changed by individuals at will. We must learn and use the rules of money to function in society. With race, societies take natural physiological differences between people and assign them social meanings that, though initially arbitrary, cannot be easily changed or thrown off by individuals. We must learn and use (at least to some degree) the rules of race to function in society. Social constructionism is a widely accepted view of race and one with which I agree.

There is, however, another view of race that has been historically important and remains relevant but rarely appears in these sorts of conversations. That is the view of race as an essential driver of history and culture, where the temperaments and talents of certain races (and the nature of interactions between different races) determine the path of human progress (or decline). This view is epitomized by British statesman Benjamin Disraeli’s 1852 statement that: “All is race. In the structure, the decay, and the development of the various families of man, the vicissitudes of history find their main solution” (Disraeli 1852, 331). Yet the view remains quite present in the background of various nativist and white nationalist movements around the globe.

There is, I would argue, a fourth way to approach the concept of race, which allows one to take seriously the second concept, social constructionism, while keeping track of (and interrogating) the important effects of the first and third concepts. Elsewhere in my work, I call this *race as technology*, but it can be more simply understood as shifting one’s focus from questions about *what race is* to analyses of *what race does* (Russell 2018).

Eric Voegelin, a German-born political theorist working in Austria during the rise of National Socialism, makes a useful distinction between *race theory* (scientific theories of race in natural science) and the *race idea* (race as a powerful political symbol used to define and shape communities) (Voegelin 1940). To paraphrase Voegelin, attempts to offer scientific theories of race persist (despite being discredited) because the sense of race as central and meaningful in our social relations endures the rise and fall of various racial theories. Thus, the essential task to undertake is to study systematically the way that the race idea operates in various contexts.

The context in which I have undertaken this work is assisted reproductive technologies. I have argued, for example, that it is less important to ask whether there is a scientific basis for the labelling of donor gametes with the self-reported racial identity of the donor than it is to explore why it is that people care so much about the supposed racial properties of donor eggs and sperm and what effect that continued caring has on our popular understandings of race and racial identities.

Similarly, while it is important for bioethicists to take up questions of access to reproductive technologies for diverse social groups, there are also crucial questions to be asked about which medical treatments and interventions get developed to treat which sorts of infertility problems and why. From a black woman’s vantage point, doctors, governments, and pharmaceutical companies seem far more interested in implanting poor women of colour with long-acting contraceptives than with preserving or restoring their fertility through accessible high-quality gynaecological care (Roberts 1999). Meanwhile, much of the fertility industry seems designed around the needs and desires of socio-economically secure white people (Ikemoto 1995).

Along the same lines, a typical division of labour would involve life scientists concerning themselves with what types of reproductive technologies *can* be developed and made safe, while bioethicists concern themselves with when and how such reproductive technologies *should* be used. I would ask that in addition to these questions, both groups consider the social and historical context in which genetically related children (or children that could pass for genetically related) are highly sought after and prized above all other forms of kinship. As Dorothy Roberts points out, “In America, whites have historically valued genetic linkages and controlled their official meaning. As the powerful class, they are the guardians of the privileges accorded to biology and they have a greater stake in maintaining the importance of genetics” (Roberts 1999, 261).

This is also the type of work I encourage bioethicists and life scientists to undertake together in a variety of contexts. I encourage them to recognize rather than deny the role that the race *idea* has played in the modern world and the development of modern sciences. I encourage them to cultivate the ability to consider things like historical context, social values, and often intangible harms to socially defined groups, even in those inquiries they might consider purely objective and scientific. I encourage them to make their own social positions as academics and researchers more transparent to themselves in order to reflect critically on their deepest background assumptions and the very framing of their research questions. In a way, Disraeli was right: Race *is* all. It structures our world, our institutions, and our thinking in ways we may never truly know but could work much harder to uncover.

## **Author Statement**

I have no financial or non-financial competing interests to declare in respect to this work. I received no financial support for this piece.
